# Data-driven translational prostate cancer research: from biomarker discovery to clinical decision

**DOI:** 10.1186/s12967-020-02281-4

**Published:** 2020-03-07

**Authors:** Yuxin Lin, Xiaojun Zhao, Zhijun Miao, Zhixin Ling, Xuedong Wei, Jinxian Pu, Jianquan Hou, Bairong Shen

**Affiliations:** 1 Department of Urology, The First Affiliated Hospital of Soochow University, Suzhou, 215006 China; 2 Department of Urology, Suzhou Dushuhu Public Hospital, Suzhou, 215123 China; 3 Institutes for Systems Genetics, West China Hospital, Sichuan University, Chengdu, 610041 China

**Keywords:** Prostate cancer, Translational informatics, Biomarker discovery, Systems medicine, Clinical application

## Abstract

Prostate cancer (PCa) is a common malignant tumor with increasing incidence and high heterogeneity among males worldwide. In the era of big data and artificial intelligence, the paradigm of biomarker discovery is shifting from traditional experimental and small data-based identification toward big data-driven and systems-level screening. Complex interactions between genetic factors and environmental effects provide opportunities for systems modeling of PCa genesis and evolution. We hereby review the current research frontiers in informatics for PCa clinical translation. First, the heterogeneity and complexity in PCa development and clinical theranostics are introduced to raise the concern for PCa systems biology studies. Then biomarkers and risk factors ranging from molecular alternations to clinical phenotype and lifestyle changes are explicated for PCa personalized management. Methodologies and applications for multi-dimensional data integration and computational modeling are discussed. The future perspectives and challenges for PCa systems medicine and holistic healthcare are finally provided.

## Background

Prostate cancer (PCa) is a malignant solid tumor commonly occurred with high incidence and mortality worldwide. According to the report of Cancer Statistics in 2019, it is the second leading cause of cancer-related death among males in the United States [1]. In Asian countries, the number of PCa patients is also increasing these years. Extensive evidences indicated that the risk of PCa is positively correlated with the age of people [2, 3], the screening of specific PCa signature is therefore of great significance for clinical decision, especially in the aging society.

Starting from normal prostate epithelium, the development of PCa is usually very slow. It may take a long time period for the growth from intraepithelial neoplasia to clinically localized PCa, and eventually progressing into the metastatic status. Unfortunately, most of the newly diagnosed PCa patients are often at the advanced stage with distant metastasis due to the limitation of typical symptoms for early detection. Although the tests of prostate-specific antigen (PSA), imaging and prostate biopsy are well conducted in PCa clinical screening, overdiagnosis and overtreatment still widely exist [4, 5]. It is generally known that the initiation and progression of PCa is extremely heterogeneous, where interactions between genetics, lifestyle and environmental factors contribute to the evolution of PCa cells [6–8]. These complex interactions bring us both opportunities and challenges for systems-level deciphering of PCa carcinogenesis and developing novel methodologies to model PCa development for biomarker discovery and clinical management [9].

The recent advances in biotechnologies and computational sciences have accumulated multi-dimensional data for PCa precision medicine and healthcare. For instance, based on next-generation sequencing the genetic architecture and gene expression patterns of normal and PCa population can be tested at low cost. The mutations and anomalous expression of genes have been witnessed to be oncogenetic or anticarcinogenic in PCa progression and invasion, which are useful for PCa risk prediction and classification [10–15]. The clinical physiological data collected from digital and smart mobile devices promote the development of preventive and participatory medicine. Using B-ultrasound, computed tomography (CT) and magnetic resonance imaging (MRI), the size and severity of tumors can be evaluated and measured in a direct way. As an emerging and interdisciplinary subject, the concept of translational informatics has been advocated for medical study. Compared with bioinformatics and biomedical informatics which mainly focus on the data at molecular and individual levels [16], translational informatics covers a broad range of topics and it integrates biomedical data including genome, transcriptome, proteome, metabolome and imaging spectrum with multi-variable social network information, such as the mechanisms from lifestyle and environmental factors positively or negatively affecting PCa [17, 18].

Systems medicine is also a data-driven paradigm for disease management. Under the framework of systems biology and medicine theory, systems medicine emphasizes the holistic properties of diseases and bridges the genotype–phenotype associations for PCa precision healthcare [19–21]. For example, the genetic screening helps the early detection of PCa risk and environmental changes regulate PCa growth through an epigenetic manner [22, 23]. Image parameters play insightful roles in PCa diagnosis and prevention from lifestyle medicine encourages the personalized treatment of PCa patients [24]. Since the complexity and variety of PCa big data, one of the key issues needed to be addressed is how to integrate the different data resources for computational modeling, and to simulate the dynamical changes during PCa development. The identification and prioritization of specific biomarkers and functionally driven players from the noisy and multi-structural data is the goal of translational informatics for PCa systems medicine, and this would promote the translation of basic biomedical research into clinical applications and decisions.

This review aims to summarize state of the art for data-driven translational prostate cancer research. First, the heterogeneity in PCa development and clinical strategies for PCa precision theranostics are briefly explicated. Then biomarkers and risk factors for PCa personalized management are sequentially introduced from three aspects, i.e., molecular alterations, clinical phenotype features, and environmental effects. The data resources, computational models available for PCa knowledge discovery and translational application are comprehensively summarized, and the latest techniques in artificial intelligence (AI) and the fifth generation (5G) mobile networks for PCa clinical practice are discussed. Future perspectives and challenges related to PCa systems medicine are provided in the end.

## Heterogeneity in PCa development and theranostics

### PCa development and evolution

**Fig. 1:**
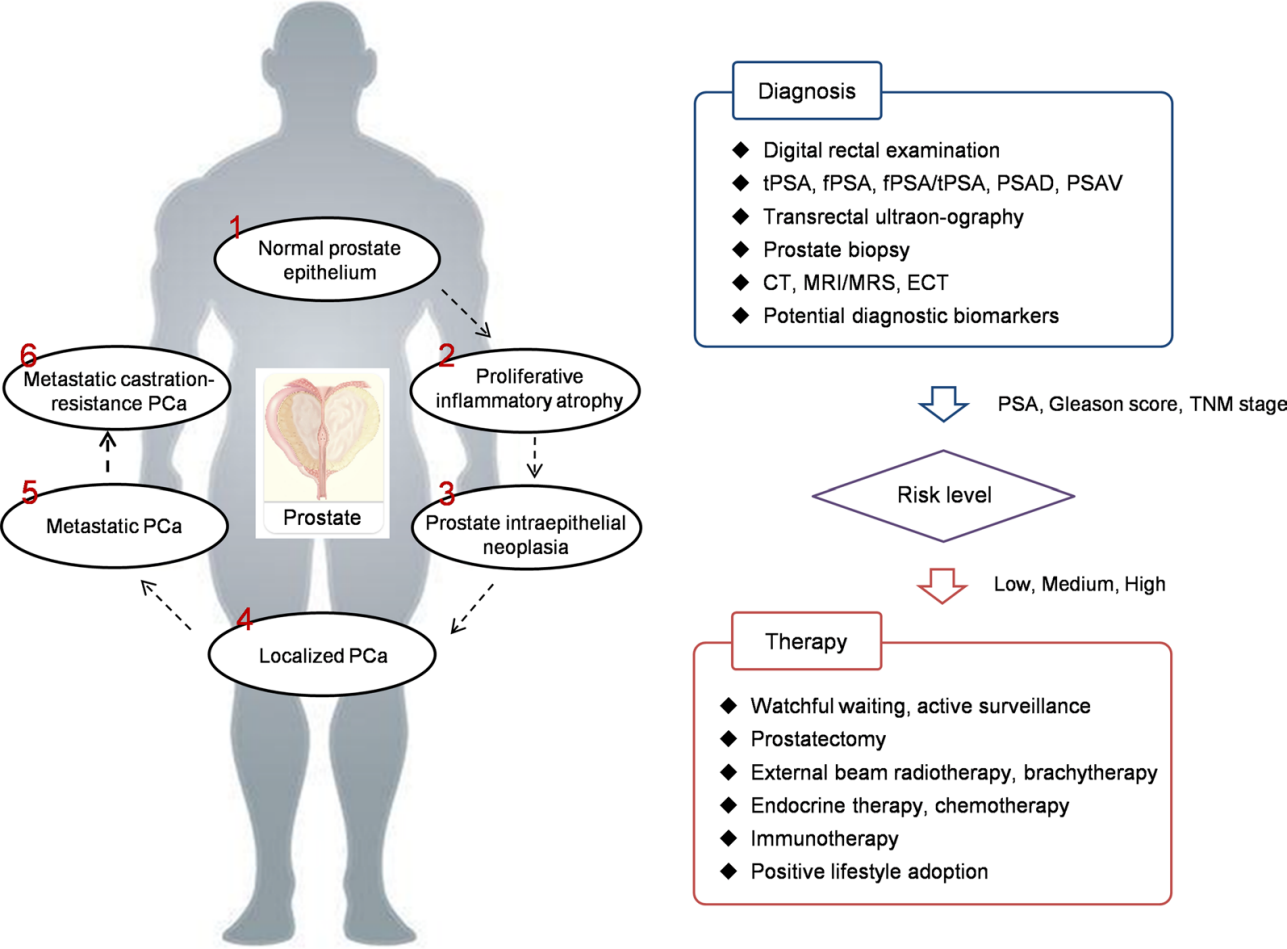
PCa development and theranostics. *PCa* prostate cancer, *PSA* prostate specific antigen, *tPSA* total PSA, *fPSA* free PSA, *PSAD* PSA density, *PSAV* PSA velocity, *CT* computed tomography, *MRI* magnetic resonance imaging, *MRS* magnetic resonance spectroscopy, *ECT* emission computed tomography, *TNM* tumor node metastasis

PCa is a kind of chronic consumptive diseases. It may take several years or even decades for the development from normal prostate tissue to malignant symptoms. However, under some circumstances it shows a high degree of invasiveness, which can be transferred to lymph nodes, bone, brain and other organs. As illustrated in Fig. 1, PCa originates in normal prostate epithelium. Based on the stress from both genetic and external environments, the out-of-control division and proliferation of tumor cells promote the formation of clinically localized PCa, and further develop into metastatic and castration-resistant states. Compared with primary localized PCa, metastatic PCa (mPCa) and metastatic castration-resistance PCa (mCRPC) have higher aggressiveness, and they always lead to poor prognosis and shorter overall survival time.

The development and evolution of PCa is a complex and heterogeneous process. For example, the incidence of PCa has obviously geographical and ethnic differences, where Western population shows a significantly higher incidence rate than that of Asian. The age, race and genetic background are risk factors of PCa. Ishak et al. [25] performed a comprehensive review on PCa susceptibility genetic variants in men with high risks based on genome-wide association studies. They found that the risk of PCa could be associated with single nucleotide polymorphisms (SNPs) in genes. In Chinese Han population, Xu et al. [26] identified two risk-associated loci for PCa on chromosomes 9q31.2 (rs817826) and 19q13.4 (rs103294), respectively. These findings improve the understanding of gene-level susceptibility heterogeneity between Chinese and Western population. In addition, extensive efforts demonstrated that the lifestyle, living environment and career of people have positive or negative effects on PCa occurrence and progression [27, 28].

### Clinical strategies for PCa diagnosis and therapy

In general, there are no typical symptoms in the early stage of PCa. With the growth of tumor, lower urinary tract symptoms will appear when the urethra and bladder have been invaded. As described in Fig. 1, there are a series of clinical strategies can be used for PCa screening, including digital rectal examination (DRE), PSA testing, transrectal ultrasonography, CT, emission computed tomography (ECT), and MRI. In particular, prostate biopsy is considered as the gold standard for PCa diagnosis. Based on the testing results, the Gleason score and tumor node metastasis (TNM) stage are defined and measured to evaluate the risk level of PCa patients.

The selection of therapeutic methods depends on the stage of PCa development. The difference between the incidence and mortality of PCa varies dramatically. According to the routine autopsy reports, approximately 60–70% of the elderly men suffer from histological PCa, but most of them are silent and have no clinical progress [29]. For patients with low PCa risk, watchful waiting and active surveillance are preferred before positive indications increased. Currently, prostatectomy is one of the most effective ways for the treatment of localized PCa, and these patients often have good prognosis. However, the survival rate and quality of life in patients with advanced mPCa are seriously reduced. Most of the patients have missed right surgical opportunity. Although endocrine-guided therapy can delay the progression of mPCa to a certain extent, many patients develop to be castration-resistant after the treatment for a period of time, and eventually move into mCRPC states. To fight against this, Docetaxel-based chemotherapy and Abiraterone-based new endocrine plans light the longing for improving the living quality. Unfortunately, patients need to balance the advantages and drawbacks among drug sensitivity, side effects and the cost.

As reported by Hanahan et al. [30], avoiding immune destruction is one of the hallmarks of cancers. Owing to the high specificity and long-lasting anti-cancer effects, immunotherapy is becoming a hot area in cancer treatment [31]. There are currently two immunotherapy options approved by Food and Drug Administration for PCa, i.e., Pembrolizumab [32] and Sipuleucel-T [33], and they have shown great significance in PCa clinical management. For translational research, Jafari et al. [34] reviewed the latest findings in the field of immune checkpoints for PCa application, such as CTLA-4, PD-1, PD-L1, B7-H3, etc., and potential biomarkers correlated with immune infiltrates in tumor microenvironment are also identified for providing candidate PCa therapeutic targets [35].

It should be noticed that the present clinical indices and therapeutic approaches still have limitations on PCa personalized medicine. For example, the specificity of serum total PSA is not powerful enough to indicate the true states of PCa development, since the benign prostatic hyperplasia and inflammation also hold the potential to increase the expression level slightly. It is generally known that PCa is a complex disease resulting from a combination of disorder between genetic and environmental factors. Hence the integration of both molecular and clinical data would contribute to the dynamical modeling of disease changes for actionable player identification, thereby bringing a systems medicine avenue toward ‘P4’, i.e., predictive, preventive, personalized and participatory, medicine spectrum in the era of translational informatics [36].

## Biomarkers and risk factors for PCa personalized management

### Susceptibility and prevention from molecular alterations

The identification of biomarkers with high sensitivity and specificity for personalized PCa management is an important direction of translational informatics studies. Compared with traditional computational framework inferring candidate players from a hypothesis-driven trail to experiment-guided validation, translational informatics learns the knowledge of diseases based on big data modeling and simulating, which increases the possibility for the holistic description of dynamical characteristics during disease development and evolution [17].

According to systems biology paradigm, biomarkers can be classified at the molecular, clinical phenotype and environmental level [37]. As listed in Table 1, accumulating studies identified that the mutation and abnormal expression of biological molecules are functional for PCa risk prediction, prognosis and targeted therapy, and some of them have been successfully applied to clinical genetic testing and auxiliary diagnosis. For example, pathogenic sequence variants in *BRCA1* and *BRCA2* are reported to be linked with PCa risk and severity. Compared with the reference bin c.1001–c.7913, Patel et al. [38] found that such variants in the 3′ region of *BRCA2* (c.7914+) had a significant association with the increased risk of PCa development, and it also held the power for aggressiveness indication. For patients with mPCa, the incidence of inherited DNA-repair gene mutations was significantly higher than that in localized PCa, and the variants in DNA-repair associated genes such as *BRCA1*, *BRCA2*, *ATM*, *CHEK2*, *RAD51D* and *PALB2* were involved in the process of PCa metastasis [39]. In addition to genetic mutations, epigenetic alteration is also reported to be associated with PCa development. For example, the methylations in *GBX2* and *CDO1*, respectively, were associated with the risk of PCa biochemical recurrence and biochemical recurrence-free survival of PCa patients [40, 41].

**Table 1:** Molecular biomarkers for PCa management

Official symbol	Type	Function	PMID
Genes			
*BRCA1*, *BRCA2*	Risk prediction	Mutations are associated with high risk of PCa development and aggressiveness	31723001
*ATM*	Risk prediction	Mutations are associated with grade reclassification and aggressive PCa	30309687
*CHEK2*, *PALB2*, *RAD51D*	Risk prediction	Germline mutations are associated with mPCa through mediating DNA-repair processes	27433846
*CDK12*	Risk prediction	Mutations are associated with high-grade PCa and the development of mPCa	31640893
*GBX2*	Prognosis	The methylation predicts higher risk of biochemical recurrence	31200836
*CDO1*	Prognosis	The promoter methylation is associated with the biochemical recurrence-free survival of PCa patients	27689475
*MLH1*, *MSH6*, *PMS2*	Prognosis	Up-regulation is associated with tumor aggressiveness and early PSA recurrence	27803051
*TP53*	Prognosis	Predicting the outcome of mCRPC patients treated with Abiraterone or Enzalutamide	30209161
*PDL1*	Prognosis	The expression is associated with high PCa risk and the biochemical recurrence of patients following adjuvant hormonal therapy	31289580
*TRIM24*	Prognosis	The expression is useful for risk stratification and recurrence evaluation	31178279
*RSPO3*	Prognosis	Lower expression is associated with PCa invasiveness and metastasis	30987640
*CRTC2*	Prognosis	Lower expression predicts worse pathologic outcomes and postoperative survival	30838340
*TMEM45B*	Prognosis	Over-expression is associated with PCa progression and metastasis	30249106
*AR*-*V7*	Therapy	The expression indicates the resistance of mCRPC patients to androgen axis-targeted therapies	31653572
*HMGB1*	Therapy	Up-regulation promotes PCa development and metastasis through Akt pathway and *BRG1*-mediated EMT	31410208
miRNAs			
*miR*-*182*-*5p*, *miR*-*375*-*3p*	Diagnosis	Circulating levels are associated with the advanced pathologic stage and metastasis	31572685
*miR*-*204*-*5p*	Diagnosis	Down-regulation predicts bone metastasis and other clinicopathological characteristics	31678733
*miR*-*1246*	Diagnosis	Down-regulation predicts PCa aggressiveness and metastasis	29437039
*miR*-*218*-*5p*	Diagnosis	Serum down-regulation is associated with bone metastasis of PCa	30870834
*miR*-*98*-*5p*, *miR*-*152*-*3p*, *miR*-*326*, *miR*-*4289*	Diagnosis	Up-regulation in plasma predicts the occurrence of PCa	30845775
*miR*-*1207*-*3p*	Prognosis	Up-regulation predicts a high risk of PCa recurrence and other clinical outcomes	27267842
*miR*-*34a*-*5p*	Prognosis	Prediction of response to docetaxel and PCa progression	25053345
*miR*-*424*-*3p*	Therapy	Low expression is associated with clinical failure in PCa	31337863
lncRNAs			
*PSLNR*	Diagnosis	Inhibiting PCa progression through the p53-dependent pathway	31269242
*PCA3*	Diagnosis	Modulating the survival of PCa cells via *AR* signaling, and the urine level is useful for PCa diagnosis	31762940
*HOTTIP*	Prognosis	Over-expression is associated with poor prognosis and clinicopathologic features	31570281
*SNHG12*	Prognosis	Predicting PCa prognosis and promoting PCa tumorigenesis through sponging *miR*-*133b*	31267540
*NAP1L6*	Prognosis	Over-expression promotes the proliferation, migration, and invasion of PCa cells, and indicates larger tumor size, distant metastasis, and shorter survival time	30154665
*TMPO*-*AS1*	Prognosis	Over-expression is associated with tumor progression and poor prognosis	30105831
*APP*	Therapy	Up-regulation promotes cell migration and invasion via sponging *miR*-*218* to facilitate the expression of *ZEB2/CDH2*	31107971
*MALAT1*	Therapy	Up-regulation is associated with proliferation and migration of PCa cells, and the silencing inhibits PCa progression	29942138
circRNAs			
*circ_0044516*	Diagnosis	Promoting cell proliferation and metastasis	31625175
*circFOXO3*	Diagnosis	Up-regulation is positively correlated with the Gleason score and PCa development	31733095
*circ_001206*	Diagnosis	Down-regulation is associated with PCa clinical features and the over-expression inhibits the proliferation, migration, and invasion of PCa cells	31198063
*circZMIZ1*	Therapy	Up-regulation promotes the proliferation of PCa cells	31686520
Metabolites			
Myo-inositol	Diagnosis	Increased level is associated with the high aggressiveness of PCa	29581441
Hyperpolarized 13C lactate	Diagnosis	Increased level predicts the presence and aggressiveness of PCa	23532911
Spermine, citrate	Diagnosis	Decreased level is associated with higher aggressiveness of PCa	23626811
Alanine, lactate	Diagnosis	Increased level is detected in PCa tissues compared with benign prostate tissues	18727052
Bone-specific alkaline phosphatase	Prognosis	Increased level is associated with bone metastases and shorter overall survival of androgen-independent PCa patients	16740758

*PCa* prostate cancer, *mPCa* metastatic prostate cancer, *mCRPC* metastatic castration resistant prostate cancer, *EMT* epithelial-mesenchymal transition, *miRNA* microRNA, *lncRNA* long non-coding RNA, *circRNA* circular RNA, *PMID* PubMed ID

Most of the studies nowadays raise the attention to non-coding RNAs (ncRNAs) in PCa carcinogenesis, including microRNAs (miRNAs), long non-coding RNAs (lncRNAs) and circular RNAs (circRNAs). Although these members do not encode proteins, they can regulate gene expression at the post-transcriptional level. In particular, lncRNAs, circRNAs and messenger RNAs (mRNAs) are known to regulate each other by competitively sharing the same miRNA response elements and finally affect the expression of down-stream genes based on competitive endogenous RNA (ceRNA) mechanisms [42]. Bidarra et al. [43] introduced that the levels of circulating *miR*-*182*-*5p* and *miR*-*375*-*3p* were candidate biomarkers to predict the advanced pathologic stage and metastasis of PCa with favorable sensitivity and specificity. Since Docetaxel-resistance limits the therapy of mCRPC patients, Corcoran et al. [44] found that *miR*-*34a* was a key player in PCa cells to regulate *BCL*-*2* and to further affect Docetaxel response. Moreover, some of the targets of this miRNAs, e.g., *SNCA*, *SCL7A5*, had strong relationships with PCa poor prognosis. As a specific lncRNA, *PCA3* was widely investigated in PCa studies. It modulated the survival of PCa cells by regulating *AR* signaling [45], and was recognized as a significant predictor for PCa grade reclassification [46]. In therapeutics, *APP* and *MALAT1* played oncogenic roles in the migration of PCa cells, where the former sponged *miR*-*218* to facilitate the expression of *ZEB2/CDH2* and the latter modulated PCa progression via the *MALAT1*-*miR*-*1*-*KRAS* axis [47, 48]. In contrast to miRNAs and lncRNAs, circRNAs are a class of ncRNAs with special circle structures. Although the functions have not been clearly deciphered, they are reported functionally important to the tumorigenesis. For example, the up-regulation of *circFOXO3* was positively correlated with the Gleason score and PCa progression level, whereas the down-expression of *circ_001206* could predict the poor clinicopathologic features for PCa patients [49, 50].

In addition to genomic, epigenomic and proteomic studies, transcriptomic and metabolomic signatures are functional in indicating the significant changes during PCa development. For example, Emami et al. [51] trained cis-regulatory models using transcriptome-wide gene expression data from PCa and normal European ancestry men. They found that the increased expression of *TMPRSS2* was associated with PCa risk and many genes showed the pattern for allele-specific transcriptional activation by PCa-associated regulators such as *AR*. Since the metabolite profiling is now widely used in screening biomarkers for cancer prediction, Kelly et al. [52] reviewed the metabolic biomarkers identified for PCa diagnosis, prognosis and treatment. Based on a replicated experiment, they found that the metabolite profiling could precisely discriminate PCa and benign patients, tumor aggressiveness, recurrence groups, and cases with good responses to therapeutics. Vandergrift et al. [53] performed a retrospective study on three groups of patients, i.e., PCa, benign prostatic hyperplasia, and the normal control, to identify metabolomic biomarkers for PCa aggressiveness prediction, and the result indicated the association between the increased myo-inositol level and highly aggressive PCa. Besides, hyperpolarized 13C lactate, spermine, and citrate are also convinced to serve as biomarkers for measuring the degree of PCa aggressiveness [54, 55].

Recent studies showed that plasmatic nanovesicles called exosomes may be highly helpful in the near future for early diagnosis and follow-up of PCa patients, because some RNAs, proteins or other molecules extracted from exosomes could serve as biomarkers for cancer detection. Logozzi et al. [56] conducted a prospective clinical study using two different methodological approaches, such as Immunocapture-based ELISA and nanoscale flow cytometry, to characterize plasmatic exosomes. They provided a clear evidence that plasmatic exosome expressing PSA could distinguish PCa patients not only from healthy individuals but also from patients affected by benign prostate hypertrophy. Due to the microenvironmental acidity of tumors, the increased plasmatic exosome expressing PSA was captured in PCa patients. In fact, culturing human PCa cells at different potential of hydrogen (pH) conditions, the result showed that low pH induced an amazing increase in ‘PSA + exosome’ release, which indicated the significance of environmental effects on biological activities [57].

With the coming age of big data, more functional molecules can be screened, and the interactions between various genetic components as well as metabolites from different sample sources will contribute to the systems understanding of PCa pathogenesis and clinical translation.

### Clinical phenotypes toward dynamical monitoring

Clinical phenotype biomarkers consist of image signatures captured by digital devices like B-ultrasound, CT, MRI, etc, special symptoms such as lower urinary tract symptoms, abdominal pain, fever and weight loss. Compared with molecular biomarkers, changes in clinical phenotypes present an intuitive picture to describe the distribution of PCa tumors, and reflect the real-time status for dynamical monitoring of PCa patients.

As shown in Table 2, there are many phenotypes can be used for PCa clinical management. Yuan et al. [58] found that zinc-specific ion chemical exchange saturation transfer MRI was a significant image factor to indicate PCa development. They supposed that the concentration of mobile zinc in healthy prostate is relatively high, so the decrease of prostate zinc content could be applied to predict the initial development of PCa. Further in vivo experiments using mouse models convinced this hypothesis [58]. Based on MRI screening, the diffusion-weighted imaging (DWI) signal is sensitive for the response of PCa bone metastasis and androgen deprivation therapy (ADT). For example, Perez-Lopez et al. [59] performed the correlation analysis between multi-parametric MRI and mCRPC bone metastases. The clinical data demonstrated that DWI signal was significantly higher in the cohort with bone metastasis. Kim et al. [60] tested the DWI signal, i.e., apparent diffusion coefficient, on PCa patients before and after ADT treatment. The result indicated the potential of DWI as a non-invasive biomarker for monitoring the dynamical changes in patients responding to ADT therapy. As an important predictor in clinical routine examination, the change in bone scan index (BSI) has been acknowledged to be powerful for measuring the prognosis and overall survival of advanced PCa patients [61–63]. In particular, it was also effective in evaluating the outcome of PCa patients under Abiraterone therapy [61].

**Table 2:** Image and clinical symptoms for PCa monitoring

Name	Type	Description	PMID
Image features			
Zinc-Specific iCEST MRI	Diagnosis	Differentiating normal and malignant prostate cells with a high fold-change	31430007
DCE-MRI	Prognosis	Reflecting PCa microcirculation and showing correlation with PSA and clinical stage	30327584
DWI signal	Prognosis	Providing information for bone metastasis and treatment of mCRPC	28906339
DWI signal	Therapy	Monitoring changes in response to androgen deprivation therapy in PCa patients	25415730
FLT PET/CT	Therapy	Predicting progression-free survival and promoting immunotherapy for mPCa patients	30700328
PSMA-derived volumetric parameters	Therapy	Evaluating whole-body tumor burden and facilitating therapy monitoring for mPCa	28522740
BSI	Prognosis	Predicting the survival time of mCRPC patients treated with Abiraterone Acetate	28723520
aBSI	Prognosis	Predicting the overall survival of patients with mCRPC	29799999
BSI, ΔBSI	Prognosis	Predicting poor overall survival and cancer specific survival	29137438
Clinical symptoms			
Lower urinary tract symptoms	Diagnosis	The symptoms increase the attention of physicians for PCa diagnosis	23313032
Lower urinary tract symptoms	Therapy	The changes are associated with the responses to salvage radiotherapy for biochemical recurrence of PCa	25991382
Urinary problems, fatigue	Therapy	Predicting health-related quality of life in PCa patients with localized radiation therapy	30263893
Sexual dysfunction	Therapy	This symptom may be caused by the therapy of M0-CRPC	28285412
Depression	Therapy	The severity is positively correlated with the duration of androgen deprivation therapy	30651026

*PCa* prostate cancer, *mPCa* metastatic prostate cancer, *mCRPC* metastatic castration resistant prostate cancer, *iCEST* ion chemical exchange saturation transfer, *MRI* magnetic resonance imaging, *DCE* dynamic contrast-enhanced, *DWI* diffusion-weighted imaging, *FLT* 3′-Deoxy-3′-18F-fluorothymidine, *PET* positron emission tomography, *CT* computed tomography, *PSMA* prostate-specific membrane antigen, *BSI* bone scan index, *aBSI* automated, BSI: ΔBSI elevated BSI change on treatment, *PMID* PubMed ID

Clinical symptoms especially the lower urinary tract symptoms provide valuable guidance for PCa diagnosis, though the specificity still needs to be investigated. Several studies focused on the urinary problems associated with the recurrence and life quality of PCa patients after radiotherapy [64–66]. Meanwhile sexual dysfunction and depression caused by different therapeutic regimes would be important for patient prognosis tracking [67, 68]. In some cases, the clinical symptom is a consequence of molecular disorders, thus the characterization of genotype–phenotype associations for PCa systems medicine is of the significance.

### Lifestyle and environmental factors for healthcare

Lifestyle is easily to be adjusted for the improvement of PCa healthcare. As described in Table 3, daily lifestyles can affect PCa development through either positive or negative ways. For example, daidzein and genistein in soy foods are protective factors for PCa [69]. The regular taking of fresh vegetables, fruits, fish, and nuts as Mediterranean diet can reduce PCa risk [70]. However, pickled vegetables, fermented soy products, salted fish, and preserved meats seem to be harmful [71]. Similar to public awareness, unhealthy habits such as tobacco consumption and alcohol intake threaten the normal function of prostate, and may increase the development of PCa based on epigenetic mechanisms [72, 73]. Interestingly, the influence of some lifestyle elements on PCa is inconsistent and controversial across different research reports. For example, Salem et al. [74] conducted a multi-center study to analyze the association between serum calcium concentration and PCa risk. They concluded that calcium was helpful for protecting against PCa. By contrast, Jackson et al. [75] found a positive linear relationship between calcium level and PCa risk, which indicated the negative effects of calcium on prostate health. To better understand the latent reasons, meta-analysis should be performed and genetic background as well as family history is urgently to be considered.

**Table 3:** Lifestyle and environmental factors for healthcare of PCa patients

Name	Type	Description	PMID
Daidzein, genistein	Positive	Soy foods and isoflavones reduce PCa risk	12869409
Toenail selenium	Positive	Higher selenium intake reduces PCa risk	14504196
Calcium	Positive	The increase of serum calcium level is associated with the decrease in PCa risk	24053657
Vitamin C	Positive	The intake is inversely correlated with PCa	11065004
Fresh fruits, vegetables, fish, nuts	Positive	Higher intake as Mediterranean diet is associated with lower the risk of PCa	28929838
Green vegetables, green tea	Positive	Regular consumption has protective effects on PCa	25256860
Tea, lycopene	Positive	Regular drinking tea and intakes of food with lycopene reduce PCa risk in Chinese men	17392149
Sleep duration	Positive	An inverse association between sleep duration and PCa risk	18542076
Smoking	Negative	Increasing the risk of high-grade PCa	25139338
Alcohol, smoking	Negative	Increasing the risk of advanced PCa	23929133
Alpha-linolenic acid, animal fat	Negative	Higher intake increases the risk of PCa	11065004
Vitamin D, calcium	Negative	Higher intake increases PCa risk in Caribbean men of African ancestry	25858172
Processed meat	Negative	Increase the risk of total PCa	17315319
Pickled vegetables, fermented soy products, salted fish, preserved meats	Negative	Higher intake increases the risk of PCa	15208621
Traffic-related air pollution, NO_2_	Negative	Exposure to ambient concentrations of NO_2_ increases the risk of PCa	23531743
Polychlorinated biphenyls	Negative	Exposure to polychlorinated biphenyls from environmental pollutants increases PCa risk	27742691
Excessive sun exposure	Negative	Excessive sun exposure increases the risk of PCa in Asians	22994730
Low pH	Negative	Low pH microenvironment is an important phenotype of PCa and other cancers	30715644

*PCa* prostate cancer, *pH* the negative of the base 10 logarithm of the molar concentration of hydrogen ions in the solution, *PMID* PubMed ID

The surrounding environment may affect the development of PCa in silence. Parent et al. [76] designed a case–control study in Montreal, and uncovered that the ground-level nitrogen dioxide from traffic-related air pollution could increase the PCa risk. Based on a combination of observational and experimental studies, Ali et al. [77] suggested that environmental polychlorinated biphenyls played a negative role in the development of high-grade PCa. Chia et al. [78] studied the Singapore PCa cases and found that excessive sun exposure could increase the risk of PCa. Last but not least, related to the pH-associated exosome release discussed above [79], low pH microenvironment is a common phenotype of all cancers [80–82]. Some preclinical reports have shown that in a model of spontaneous PCa simply administering buffers or even alkalinizing water could prevent the formation of PCa [83, 84], which brings a new vision for clinical management and holistic healthcare of PCa patients.

In recent years, more attention has been paid to develop healthy lifestyle habits and living environments for disease prevention and healthcare. To unravel the deep linkage between lifestyle, environment and PCa pathogenesis, exclusive use of single-level biomedical data is inadequate, molecular resources together with the information from social networks and population studies are crucial for pluralistic integration [17].

## Translational PCa research in big biomedical data era

### Standardization and integration of data resources for knowledge discovery

In big biomedical data era, the standardization and integration of information from different resources is the first step toward systems modeling and knowledge discovery. In fact, the types of PCa data are diverse [85], including: (1) molecular data regarding to the genetic structure and metabolic process of PCa patients, such as mutations, abnormal expression and dysfunctional regulation of genes, RNAs, proteins and metabolites; (2) image data screened by B-ultrasound, CT, MRI and other digitized techniques. These data provide a clear map for personalized PCa signal identification; (3) demographic and clinical phenotype data collected from electronic medical records, laboratory tests and the history of diagnosis and therapeutics; (4) lifestyle and environmental data associated with the diet, physical sports, profession, hobby interests, and surrounding environments of PCa patients.

As illustrated in Table 4, it is encouraging that a large number of databases and knowledge bases have been designed for PCa translational studies. For example, The Dragon Database of Genes associated with Prostate Cancer (DDPC) and Prostate cancer proteomics (PCP) database, respectively, increase the convenience for PCa genomics and proteomics studies [86, 87]. Reznik et al. [88] analyzed metabolomics data from tissue samples of seven cancers and developed a large-scale online resource for investigating the change in metabolite levels across cancers. At the image and clinical level, The Cancer Imaging Archive (TCIA) provides a platform for the advanced medical imaging of PCa and other types of cancer [89]. Chen et al. [90] developed the first lifestyle database (PCaLiStDB) for PCa prevention. In the current version, a total of 2290 lifestyles were manually collected via PubMed literature mining, and the related genes, biochemical indexes as well as drug responses were reasonably annotated for the lifestyle-wide association studies of PCa. In addition, Cancer of the Prostate Strategic Urologic Research Endeavor (CaPSURE) is a specific database focusing on documenting the impact of PCa, and information including clinical outcomes and health-related quality of life can be recorded [91]. To promote a standardized landscape of PCa, a data resource called Prostate Cancer Ontology (PCaO) was built by referencing the authoritative clinical guidelines to extract the core concepts associated with PCa evolution, including 637 main classes and more than 2000 synonyms (http://pcaontology.net/).

**Table 4:** Data resources and models for translational PCa informatics

Name	Description	PMID
Databases and knowledge bases		
DDPC	Dragon Database of Genes associated with Prostate Cancer: an integrated knowledge base of genes experimentally supported for PCa http://cbrc.kaust.edu.sa/ddpc/	20880996
PCP	Prostate cancer proteomics: a database created by the results from proteomic studies of human PCa http://ef.inbi.ras.ru/	22649669
Pancanmet	Pan-cancer metabolites: A web resource for metabolites and associated clinical significance across cancers http://www.sanderlab.org/pancanmet/	29396322
TCIA	The Cancer Imaging Archive: a data platform for advanced medical imaging of cancers https://www.cancerimagingarchive.net/	23884657
PCaLiStDB	Prostate Cancer Lifestyle Database: a database for lifestyle-wide association studies of PCa http://www.sysbio.org.cn/pcalistdb/	31950190
CaPSURE	Cancer of the Prostate Strategic Urologic Research Endeavor: documenting the impact of PCa, including resource utilization, clinical outcomes, health-related quality of life, etc.	8911524
PCaO	Prostate Cancer Ontology: a data resource for providing the standardized concepts and knowledge for PCa precision study http://pcaontology.net/	N/A
Computational models and tools		
PPI-based network model	miRNAs regulating hub genes in PPI network are identified for predicting high-grade PCa	30367364
PCa-specific PPI network model	Identifying module biomarkers for PCa progression based on gene expression and PPI data http://www.ibio-cn.org/HPC-PPIN/	25080090
Network vulnerability-based model	miRNAs with stronger single-line regulatory power are identified for predicting PCa metastasis http://sysbio.suda.edu.cn/MiRNA-BD/	29784056
WGCNA-based model	Identifying lincRNA module biomarkers for PCa diagnosis and prognosis	26100580
RF-based classification model	Predicting aggressive behavior of PCa based on DNA methylation data and RF modeling	31640781
CCI-reinforced SVM and RF models	Predicting the recurrence-associated death from localized PCa	31428684
CNN-based model	A computer-aided diagnosis system for automatically detecting PCa using MRI data	29772101
Deep learning model	Dividing patients with PCa into clinically meaningful groups	31238766
Integrated network and SVM model	Prioritizing PCa-associated miRNAs based on miRNA target-dysregulated network analysis and SVM classification	21768329

*PCa* prostate cancer, *RF* random forest, *SVM* support vector machine, *CCI* Charlson comorbidity index, *CNN* convolutional neural network, *MRI* magnetic resonance imaging, *PPI* protein–protein interaction, *WGCNA* weighted gene co-expression network analysis, *N/A* not applicable, *URL* uniform resource locator, *PMID* PubMed ID

Although the resources are abundant and publicly applicable, the standardization should be carefully considered before data integration and characterization since the formats of data types are highly heterogeneous. For example, the gene expression profiles are screened and calculated with different output formats, based on the experimental instruments and equipment used. Compared to the data with structural contexts, the image and environmental data on different biological layers are hard to be quantitatively measured. As noticed, the lack of standardized rules for multi-data organization limits the procedure of clinical translation. Moreover, data sharing for collaborative research is still a typical obstacle for nation-wide and multi-center clinical evaluation, and efforts need to protect the privacy and security of patient personal information. Systems medicine highlights the global and dynamical properties hidden in data. Many studies isolate the underlying interactions among different meta-resources, and solely emphasis the static patterns for further analysis. For translational applications, the integration of cross-level data in a continuous series of points would be important for the real-time monitoring and precision healthcare of PCa patients.

### Computer-aided modeling and simulating for translational applications

**Fig. 2:**
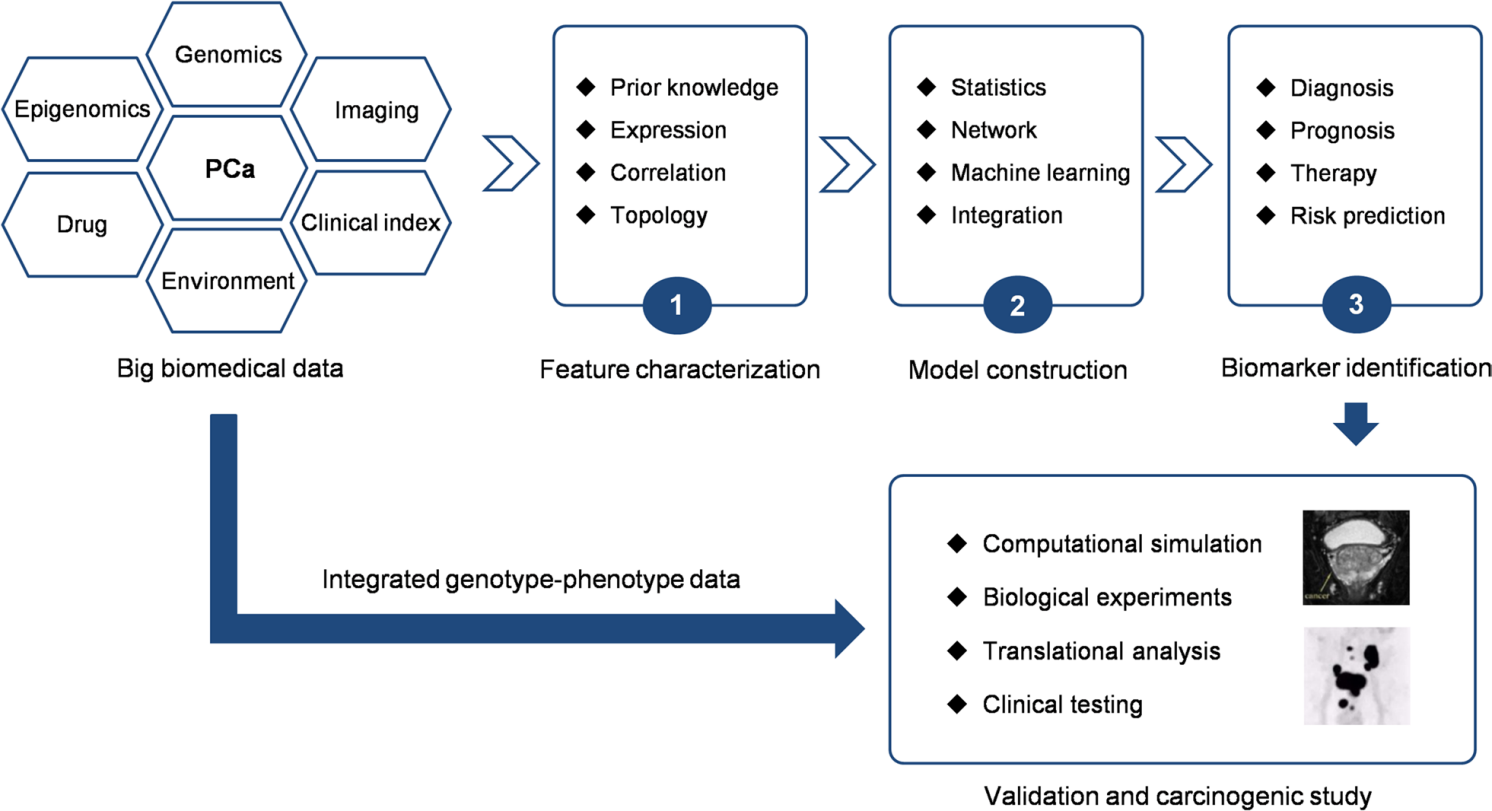
Informatics for PCa biomarker discovery and carcinogenesis analysis. *PCa* prostate cancer

With the accumulation of biomedical data, computer-aided modeling and simulating for the complex PCa process is now becoming available. As described in Fig. 2, multi-level PCa data drive the development of computational models from evidence-based feature characterization to mechanism-guided biomarker discovery. Three approaches, i.e., statistics, network modeling, and machine learning, have been widely applied to screen key players for personalized PCa management. Validation and carcinogenic studies are performed for translational analysis afterwards.

Biological networks are powerful for measuring the potential relationship among different molecular elements. As shown in Table 4, topological patterns hidden in protein–protein interaction (PPI) network, miRNA-mRNA regulatory network and gene co-expression network contribute to the implementation of PCa bioinformatics modeling. For example, Foj et al. [92] performed the differential expression analysis on miRNA and mRNA datasets downloaded from Gene Expression Omnibus (GEO) database. Based on PPI network information and miRNA-target prediction, a total of 12 miRNAs regulating no less than two of the 10 hub genes in PPI network were identified as candidate signature for high-grade (Gleason score ≥ 7) PCa detection. According to the pathway enrichment analysis, the 10 hub genes were closely associated with *PI3K*-*Akt* signaling, which was involved in the process of cell proliferation and survival. The identified miRNAs, therefore, were inferred to be functional in PCa invasion and aggressiveness. Compared with this method, Li et al. [93] integrated the gene expression data and PPI network for PCa-associated gene module biomarker identification. The difference is that the model directly mapped the transformed expression value of genes onto PPI, and extracted sub-networks specific to PCa progression based on greedy algorithm. Finally, top-ranked sub-networks identified from each PCa dataset were merged as module biomarker for PCa screening. Further enrichment analysis convinced the role of genes in *AR* nuclear signaling and *EGFR* pathway. Since miRNAs and lncRNAs are important post-transcriptional regulators, Lin et al. [94] focused on special regulatory structures in human miRNA-mRNA network, and defined the single-line regulation of miRNAs for biomarker characterization. Combined with the function of targeted genes, five miRNAs, i.e., *miR*-*101*-*3p*, *miR*-*145*-*5p*, *miR*-*204*-*5p*, *miR*-*198* and *miR*-*152*, were identified as candidate biomarkers for PCa metastasis. Cui et al. [95] applied the weighted gene co-expression network analysis to cluster lncRNAs as module biomarkers for PCa diagnosis and prognosis. The correlation between the principle component of each identified module and PCa phenotype convinced the predictive power of the lncRNAs.

In addition to network-based methods, machine learning models such as support vector machine (SVM), random forest (RF), convolutional neural network (CNN) and deep learning are used for predicting the behavior of PCa. For example, Toth et al. [96] presented a RF model to predict the aggressiveness of PCa. The DNA methylation data in PCa cohorts with good or poor prognosis were selected as the input, and genes were ranked for evaluating the relevance between the loss of methylation in partially methylated domain regions and PCa progression. Lin et al. [97] integrated the RF with SVM and Cox proportional hazard model, and developed a Charlson comorbidity index-reinforced computational framework for predicting the recurrence-related death of localized PCa. To analyze the MRI data, Ishioka et al. [98] used a CCN algorithm for automated detection of PCa cases. Eminaga et al. [99] introduced an approach for dividing PCa patients into clinically meaningful groups based on deep learning. The result demonstrated its classification ability and clinical decision possibility. Xu et al. [100] decoded crucial clues from network topology and SVM model, and prioritized candidate miRNAs for PCa diagnosis and tumorigenesis analysis.

Considering the methods for PCa translational studies, few of them uncovered the holistic features for knowledge discovery and model training. The development of PCa is complex and dynamical, thus the use of small discrete data would limit the generalization and robustness of proposed models in a big testing space. To achieve better understandings, the traditional paradigm for biomarker identification from a small set of data should be shifted into the mode of big data-based systems modeling [17]. Meanwhile, integration of molecular genotype with clinical phenotype data is essential for the translation of informatics to practical use.

### AI-based and 5G-supported clinical decision making

**Fig. 3:**
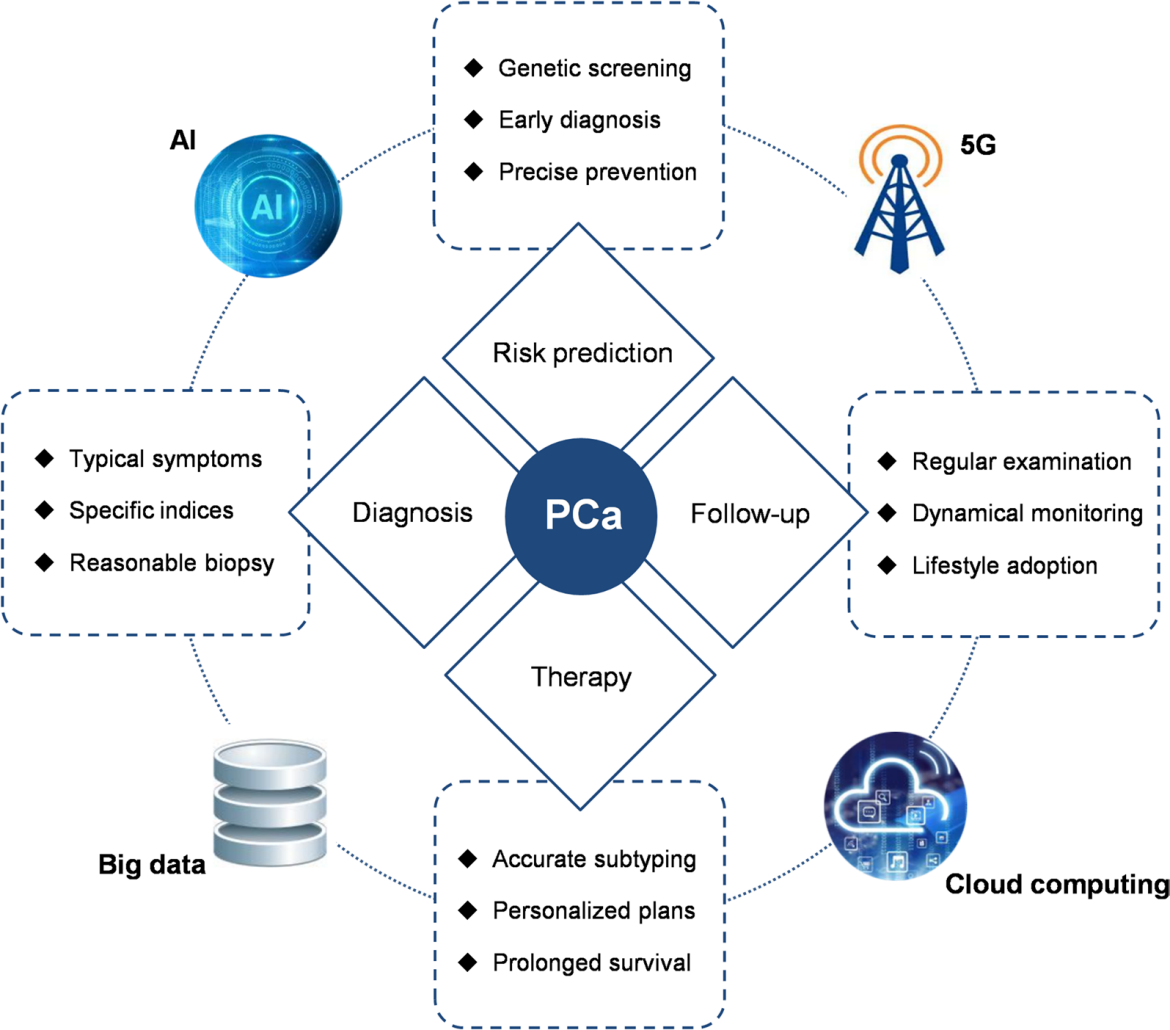
PCa holistic healthcare based on AI and 5G technology. *PCa* prostate cancer, *AI* artificial intelligence, *5G* the fifth generation mobile networks

As shown in Fig. 3, clinical management of PCa still faces difficulties in terms of the risk prediction, diagnosis, therapy and follow-up. For example, over-diagnosis and over-treatment are concerned by the public all the time due to the limitation in current indices clinically used. Prostate biopsy and prostatectomy, respectively, are the most effective way for PCa screening and treatment, however, they are both invasive, and should not be inappropriately performed on patients without carefully considering their personalized disease history.

With the rapid progress in computer sciences, AI is now changing social lives in many ways, including the medicine. As a machine with strong ability for intelligent decision, AI is able to carry out cognitive tasks based on continuous learning and refining specific skills and knowledge from provided big data. The development of machine learning algorithms and advanced image processing systems using cloud storage and computing platforms contributes to the training of computational models to automatically conduct complex studies in molecular testing, medical imaging and translational informatics, thereby improving the accuracy and clinical workflow of PCa diagnosis and therapy [101, 102]. In particular, the application of AI to screen tiny lesions from image data for early detection of digital pathology enables the advanced characterization of PCa development based on a combinative assessment of radiology and pathology [103]. In therapy, radical radiotherapy offers the long-term outcomes for high-risk PCa patients. However, the use of radiotherapy especially the whole-pelvis radiotherapy (WPRT) remains controversial due to the noise in the mixed data and some patients may not eventually benefit from such therapeutic effects. Based on the comparison and training of big data from thousands of patients treated with WPRT, AI identifies key disease regions for WPRT optimization, and improves the effectiveness and safety of pelvic radiotherapy [104]. To achieve better performance, AI techniques need to be constantly strengthened using large-scale clinical data. Nir et al. [105] compared cross-validation approaches in AI for PCa grading from digitized histopathologic images. They found that patient-based cross-validation and multi-expert evaluation could reduce the biases of AI classifiers and improve the accuracy of estimation in contrast to patch-wise cross-validation and single-expert training.

The newly developed 5G technology aims to build a network interconnection of all things and it holds the promise for high performance computing of multi-dimensional biomedical data to clinical translation. With the support of 5G, remote medical treatment has become applicable. For example, the remote surgeries can be precisely conducted by surgeons in other locations since 5G makes high-resolution videos and AI-assisted decisions with no time-lag possible. Moreover the integrated framework of ‘AI + 5G’ promotes novel medical modes for holistic health administration, such as patient engagement, mobile health, and Internet hospital, which contribute to the realization and boom of medical diversity.

## Perspectives and challenges on PCa systems medicine

**Fig. 4:**
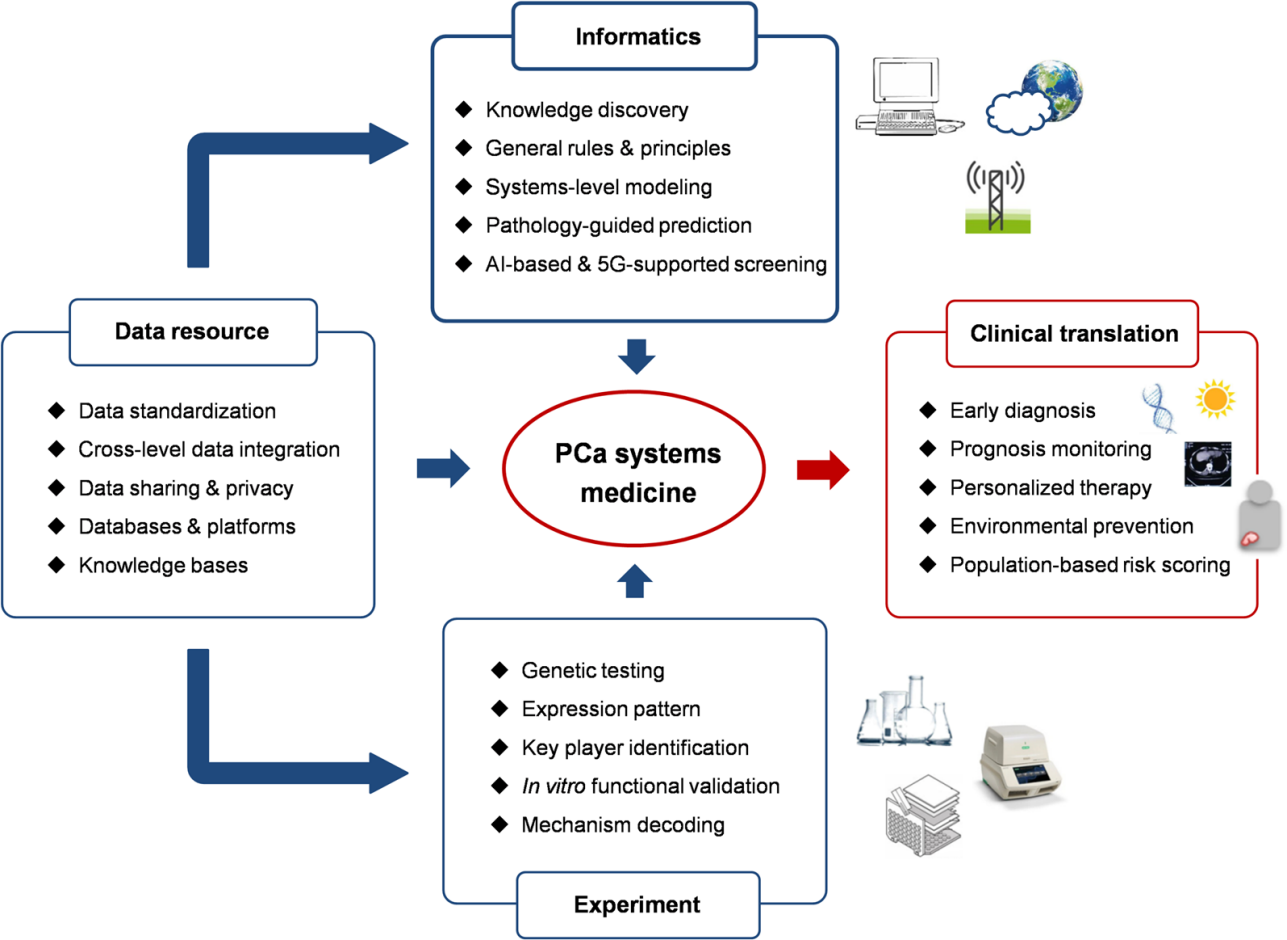
The paradigm of PCa systems medicine for clinical translation and application. The big biomedical data with multi-dimensional characteristics is the driving force for systems-based informatics modeling and experimental validation. *PCa* prostate cancer, *AI* artificial intelligence

The term of big data and translational informatics creates an unprecedented chance for PCa systems medicine, as illustrated in Fig. 4. The concept of ‘systems’ mainly covers two aspects of contexts: first, identifying and characterizing PCa signatures and carcinogenesis at the systems or network level; second, integrating molecular testing, image screening and lifestyle prevention for PCa precision medicine and holistic healthcare based both on computational and experimental techniques [106]. To achieve this goal, perspectives and challenges are needed to be concerned.

### Perspective and challenge 1: standardizing and integrating cross-level biomedical data for database construction and systems analysis

It should be admitted that data resources are the foundation and driving force for computational modeling and experimental validating of complex status associated with PCa evolution. Standardization and integration of big biomedical data for database construction and systems analysis are important for knowledge discovery and key player identification. On one hand, the development of ontology is an essential step for PCa standardized and overall description. On the other hand, data cleaning needs to be performed before the integration and application of noisy data. In addition, data sharing from multi-center studies is advocated under the promise of protecting the personal information of PCa patients [102].

### Perspective and challenge 2: proposing principles and general rules for PCa feature characterization and dynamical modeling

Until now, principles and general rules are limited for PCa feature characterization and dynamical modeling from a holistic perspective. For example, most of the studies only take the conventional parameters into account and ignore the hidden structures within the data. Systems-guided screening of biomarkers and risk factors provides functional insights in PCa pathogenesis and heterogeneity. However, it is still a challenge for translational informatics because of the considerable data sample and complexity in computer modeling. Compared with traditional modes for PCa analysis, systems biology emphasizes the identification of PCa-related genes, RNAs, proteins, pathways and non-genetic factors under an interactive and dynamical framework. The computational approaches should not only focus on the genotype changes in PCa patients, phenotype indices and clinical physiological signals are also significant for model training and testing.

### Perspective and challenge 3: integrating computational and experimental methods for population-based validation and refining the models based on big data training

Traditional methods used for biomarker validation usually start from the evaluation of candidate molecules through computational simulation, followed by biological experiments using cell lines, model animals and human prostate samples for differential expression analysis. Finally, molecules with significant expression-level alternations are selected for carcinogenesis decoding. Serving for clinical use is the ultimate aim of translational studies, however, informatics models often fails in the last phase of trials because the features previously obtained do not overcome the fact of clinical complexity and diversity [107]. Hence population-based validation ranging from a small set of subjects to big groups of patients may help refine the models, and the models in turn need the big data for continuously training and learning.

### Perspective and challenge 4: developing potential scenarios and practical standards for translational assessment and clinical utility of identified biomarkers

Although biomarkers ranging from molecular alternations to clinical phenotype changes are accumulated in PCa studies, the efficiency and precision still need to be considered for translational application. Currently, a growing number of biomarkers have been identified and shown the performance on PCa risk stratification in vitro, however, most of them tend to have low quality and their value should be carefully validated and documented in clinical utility. To achieve better outcomes and indicate the complex heterogenicity during PCa evolution, practical standards are necessary to be provided for biomarker comparison and moreover, the question regarding the optimal combination of biomarkers with other clinical indices is expected to be defined and answered [108].

Owing to the perspectives on potential risk screening and personalized healthcare, translational informatics would be a powerful tool against PCa in future clinical practice. The existed challenges encourage the development of novel theories and technologies for PCa precision medicine, and contribute to the transition from PCa management toward ‘P4’ continuum for systems health promoting [109, 110].

## Conclusion

In this review, the recent progress and future perspective in translational informatics for PCa systems medicine are comprehensively introduced and discussed. In the era of big biomedical data, computer-aided biomarker discovery ranging from genetic factors to environmental changes gives insights in PCa heterogeneity understanding and contributes to precision medicine and holistic healthcare of PCa patients.

## Data Availability

The data generated or analyzed during this study are available from the corresponding author upon reasonable request.

## Abbreviations

PCa: Prostate cancer
PSA: Prostate-specific antigen
CT: Computed tomography
MRI: Magnetic resonance imaging
AI: Artificial intelligence
5G: The fifth generation mobile networks
mPCa: Metastatic prostate cancer
mCRPC: Metastatic castration-resistance prostate cancer
SNP: Single nucleotide polymorphism
DRE: Digital rectal examination
ECT: Emission computed tomography
TNM: Tumor node metastasis
ncRNA: Non-coding RNA
miRNA: microRNA
lncRNA: Long non-coding RNA
circRNA: Circular RNA
mRNA: Messenger RNA
ceRNA: Competitive endogenous RNA
DWI: Diffusion weighted imaging
ADT: Androgen deprivation therapy
GEO: Gene expression omnibus
DDPC: The dragon database of genes associated with prostate cancer
PCP: Prostate cancer proteomics
pH: The negative of the base 10 logarithm of the molar concentration of hydrogen ions in the solution
TCIA: The cancer imaging archive
PCaLiStDB: Prostate cancer lifestyle database
CaPSURE: Cancer of the prostate strategic urologic research endeavor
PCaO: Prostate cancer ontology
PPI: Protein-protein interaction
SVM: Support vector machine
RF: Random forest
CNN: Convolutional neural network
WPRT: Whole-pelvis radiotherapy
